# Comprehensive Driving Assessment to Enable Driving Resumption in Adults Recovering From Critical Illness: A Prospective Cohort Study

**DOI:** 10.1097/CCM.0000000000007316

**Published:** 2026-09-11

**Authors:** Chloe Apps, Kate Brooks, Gillian Radcliffe, Kieran Mahon, Patricia Helen Morris, Gavin Saunders, Michelle Giles, Leah Homden, Jessica Gregory, Paula O’Shea, Melanie Gager, Liza Keating, Amy Grimadell-Jones, Lucy Pearson, Barnaby Sanderson, Andrew Slack, Helen Vollmer, Katie Susser, Manu Shankar-Hari, Natalie Pattison, Louise Rose, Joel Meyer

**Affiliations:** 1 Guy’s and St. Thomas’ NHS Foundation Trust, London, United Kingdom.; 2 King’s College London, London, United Kingdom.; 3 QEF Mobility Services, Surrey, United Kingdom.; 4 University of Melbourne, Melbourne, VIC, Australia.; 5 Epsom and St Helier University Hospitals NHS Trust, Surrey, United Kingdom.; 6 St George’s University Hospitals NHS Foundation Trust, London, United Kingdom.; 7 Royal Berkshire NHS Foundation Trust, Reading, United Kingdom.; 8 Surrey and Sussex Healthcare NHS Trust, Surrey, United Kingdom.; 9 University of Hertfordshire, Hertfordshire, United Kingdom.; 10 East and North Herts NHS Trust, Stevenage, United Kingdom.

**Keywords:** cohort, driving, intensive care, post-intensive care syndrome, recovery

## Abstract

**OBJECTIVES:**

Primary objective: To determine the driving ability of adult ICU survivors 2 months after hospital discharge. Secondary objectives: Evaluate driving resumption at 3, 6, and 12 months; factors associated with driving resumption; and acceptability of a comprehensive driving assessment.

**DESIGN:**

Observational cohort study.

**SETTING:**

Certified driving assessment center in the United Kingdom.

**PATIENTS:**

Adult ICU survivors who received mechanical ventilation (minimum 72 hr) and/or extracorporeal membrane oxygenation.

**INTERVENTIONS:**

A Driver Vehicle Licensing Agency-approved, driving ability assessment comprising physical and cognitive evaluation, and practical driving skills using a driving simulator, private track in-car assessment, and on-road in-car assessment.

**MEASUREMENTS AND MAIN RESULTS:**

We recruited 40 participants (median age, 53 yr [interquartile range (IQR), 46–64 yr]; 13 [32%] female) from six hospitals. Of these, 33 completed the driving assessment at a median of 12.5 weeks following hospital discharge. Twenty-six participants (79%) were assessed as capable of driving a standard vehicle; however, only 15 (45%) had resumed driving. Seven participants (7/33, 21%) were deemed unable to drive a standard vehicle due to either physical (*n* = 4/7, 57%), psychological (*n* = 2/7, 29%), or cognitive impairment (*n* = 1/7, 14%). Driving resumption following adaptations, retraining, or refresher lessons occurred in four participants (*n* = 4/7, 57%) in the 12-month study period. Self-reported driving resumption at 3, 6, and 12 months was observed in 21 (62%), 28 (82%), and 30 (91%) participants, respectively. Time to driving resumption ranged from 7 to 274 days following hospital discharge. Median (IQR) driving confidence (10-point Likert scale) increased from 7 (5–8) pre-assessment to 9 (7–10) post assessment (*p* < 0.05). Driving assessment acceptability was rated very good or excellent by 32 participants (97%).

**CONCLUSIONS:**

Using a comprehensive driving assessment 3 months following hospital discharge, approximately three quarters of this cohort were deemed able to drive a standard vehicle, yet less than half had resumed driving. Of those with a driving-related impairment, most could resume driving following intervention.

**KEY POINTS**

**Question**: How is driving ability as assessed by a comprehensive driving assessment affected by critical illness and what are the rates of driving resumption in adults recovering from critical illness?

**Findings**: In this observational cohort study of critical illness survivors, 33 participants completed a comprehensive driving assessment with 26 (79%) being deemed able to drive a standard vehicle, yet only 15 (45%) had resumed driving. The other seven participants (21%) were deemed unable due to a physical (*n* = 4, 57%), psychological (*n* = 2, 29%), or cognitive impairment (*n* = 1, 14%). Of these seven, four (57%) successfully resumed driving following adaptations, retraining, or refresher lessons.

**Meaning**: Formal comprehensive driving assessment of critical illness survivors 2 months after hospital discharge identified avoidable driving delay and detected driving-related physical, psychological, and cognitive impairments that could mostly be overcome.

Worldwide, the incidence of critical illness is estimated to be 30–45 million per year (1). With over 80% surviving to hospital discharge, many of these individuals experience prolonged (or incomplete) recovery due to the combination of physical, psychological, and cognitive deficits (termed post-intensive care syndrome) (2). These deficits influence return to daily living and vocational occupations. Driving a vehicle, required by many to resume these activities, is a complex task that requires adequate physical, sensory, psychological, and cognitive functioning (3). Therefore, functional, physical, and cognitive challenges experienced by critical illness survivors have the potential to impact driving ability.

Inability to return to driving adversely affects independence, ability to access healthcare and community services, return to vocational occupations, such as work, study, or volunteering, health-related quality of life, and social role performance (4–6). However, driving status is often neglected in clinical conversations, resulting in financial, social, and psychological cost to the patient (5). Furthermore, only a small number of studies explore driving ability after critical illness. These studies report substantial delays in driving resumption (4, 7, 8).

Studies conducted on driving resumption to-date are mostly retrospective and limited to patient self-reported driving status and/or driving-related behaviors (4, 5, 7, 9, 10). A systematic review including 19 studies on driving resumption after acute illness (only two pertained specifically to critical illness survivors) highlighted a lack of evidence to inform clinical recommendations (11). Furthermore, driving license regulatory bodies (e.g., Driver Vehicle Licensing Agency [DVLA], Association of the Australian and New Zealand Transport Agencies [Austroads], Canadian Medical Association, or U.S. National Highway Traffic Safety Administration) do not provide guidance specific to post-ICU deficits (12–15). This evidence gap, lack of guidance, and absence of a standard pathway for assessing driving fitness present a challenge to clinicians when making recommendations relating to driving resumption, and to patients and families who are trying to understand associated risks.

Our overall aim was to prospectively and objectively evaluate driving ability, driving resumption, and driving-related impairments in critical illness survivors. The primary study objective was to quantify driving ability using a comprehensive driving assessment in individuals recovering from critical illness conducted 2 months after hospital discharge. Secondary objectives were to describe participant reported time to driving resumption at 3, 6, and 12 months, factors associated with driving outcome, and perceived acceptability of the comprehensive driving assessment.

## MATERIALS AND METHODS

### Study Design and Setting

We undertook an observational cohort study that incorporated a comprehensive driving assessment at a certified driving assessment center with participants recruited from six U.K. hospitals providing post-ICU recovery services. Potential participants were screened for eligibility on ICU discharge and approached for consent before hospital discharge or after discharge home. Recruitment commenced in February 2020, paused during the 2020–2021 waves of the pandemic, and closed in September 2024.

### Participants

Our inclusion criteria comprised: 1) adults who received mechanical ventilation and/or extracorporeal membrane oxygenation for greater than or equal to 72 hours, 2) held a driving license and drove a car in the 12 months before hospital admission, 3) intended to resume or had already resumed driving, 4) able and willing to travel to the driving assessment center (travel costs covered by the study), and 5) provided informed consent.

Exclusion criteria were: 1) driving ineligibility (as per DVLA medical guidance [16]), 2) invalid, revoked or expired driving license, 3) deemed unlikely to return to independent living status, 4) high risk of illness recurrence or hospital readmission as determined by the research team from review of the clinical record and clinical trajectory, and 5) psychosocial circumstances deemed likely to adversely affect study participation (including adverse health of family member and recent bereavement).

### Study Interventions

Participants underwent a comprehensive driving ability assessment at a Driving Mobility accredited assessment center 2 months after discharge from hospital, or a rehabilitation center, to home. The driving assessment comprised a comprehensive review of driving ability consisting of a participant interview, physical assessment, cognitive assessment, assessment on a driving simulator, and an in-car assessment. The assessment was completed jointly by an occupational therapist (OT) and a specialist approved driving instructor with expertise in driving assessment and resumption, both employed by the driving center. Physical, cognitive, driving simulator, in-car, and on-road assessment components were completed as indicated, based on professional judgment by the assessing team.

The physical assessment comprised range of movement, muscle strength, and coordination testing. The cognitive battery was based on the Rookwood Driving Battery and evaluated executive functioning and perceptual analysis (**Supplementary Material 1**, https://links.lww.com/CCM/I44) (17). It has been validated in neuropsychiatric populations including dementia, cerebrovascular accident, and traumatic brain injury (18, 19).

The driving simulator provided measurements of driving characteristics including steering ability, brake force, reaction time, and decision-making times (20). Information on these measurements and scoring values are shown in **Supplementary Material 2** (https://links.lww.com/CCM/I44). Driving control adaptations, including hand controls, left foot brake and accelerator, and steering wheel aids, were trialed during assessments when deemed indicated.

For the in-car assessment, participants were provided with a dual-controlled vehicle like the usual vehicle they drove or, where informed by the preceding assessment, a vehicle with adaptations meeting their needs. The private track at the driving center has features of a normal road including traffic lights and give way areas with incline and flat segments. The on-road assessment was conducted on public roads including single lane roads and dual carriage ways (divided highways or freeways) with speed limits between 20 and 60 miles/30–100 kilometers per hour. Each drive was monitored for safety with participants evaluated on the use of eight practical skills (accelerator, gears, mirrors, brake, parking brake, secondary controls, steering, and clutch if driving a manual vehicle) and seven higher driving skills (attention, speed of processing, executive function, sequencing and coordination, memory, planning, and visuospatial perception). Each practical and higher driving skill was scored from 1 to 4 by a qualified driving instructor (1 = good, 2 = acceptable, 3 = poor, and 4 = high risk). Scores of 1 and 2 represent safe driving.

Before and immediately following the comprehensive driving assessment, participants completed a bespoke paper-based questionnaire assessing acceptability, experience to date of driving resumption (or not), and factors they perceived as associated with driving outcomes. Questionnaires included open and closed questions. A 10-point numerical Likert scale was used to assess confidence pre- and post-driving assessment. A 5-point Likert scale was used to assess acceptability. Questionnaires were developed iteratively by the research team and were assessed for face and content validity by three researchers (J.M., C.A., G.R.). We contacted participants 3, 6, and 12 months after hospital discharge to ascertain driving resumption status (and estimated date of driving resumption if applicable).

### Ethics

Ethical approval for the study was granted by the U.K. Health Research Authority and the London–West London and Gene Therapy Advisory Committee Research Ethics Committee (Study title: Feasibility of a Comprehensive Driving Assessment to Inform Driving Resumption Following Critical Illness: An Interventional Cohort Study, Research Ethics Committee Reference: 19/LO/1665, Integrated Research Application System number: 262986, approved on January 28, 2020). All procedures were followed in accordance with the ethical standards of the Health Research Authority and Research Ethics Committee and with the Helsinki Declaration of 1975. Written informed consent was obtained from all participants.

### Data Analysis

Continuous data were assessed for normality using the Shapiro-Wilk test and are presented as medians and interquartile range (IQR). Categorical data are presented as counts and proportions. We generated a Kaplan-Meier curve to examine time to driving resumption. Wilcoxon signed-rank test was used to assess change in driving confidence scores pre- and post-driving assessment. Analyses were performed using IBM SPSS Statistics Version 29.0.2.0 (IBM Corporation, Armonk, NY) (20). Tests were two-tailed with a *p* value of less than 0.05 considered statistically significant. Qualitative data from open-ended questions were analyzed deductively using content analysis, which was informed by previous research findings (21).

## RESULTS

We recruited and consented 40 participants with 33 completing all study requirements including the comprehensive driving assessment. One additional participant provided baseline and follow-up study data but did not complete the driving assessment; six withdrew. Participant flow is shown in the Consolidated Standards of Reporting Trials diagram (**Fig. 1**) (22). Of eligible patients, approached, 81 (49%) declined with most frequently cited reasons being: 1) no perceived need of assessment (*n* = 26, 32%); 2) ongoing health concerns (*n* = 12, 15%); and 3) unable to meet study commitments (*n* = 11, 14%).

**Figure 1.**
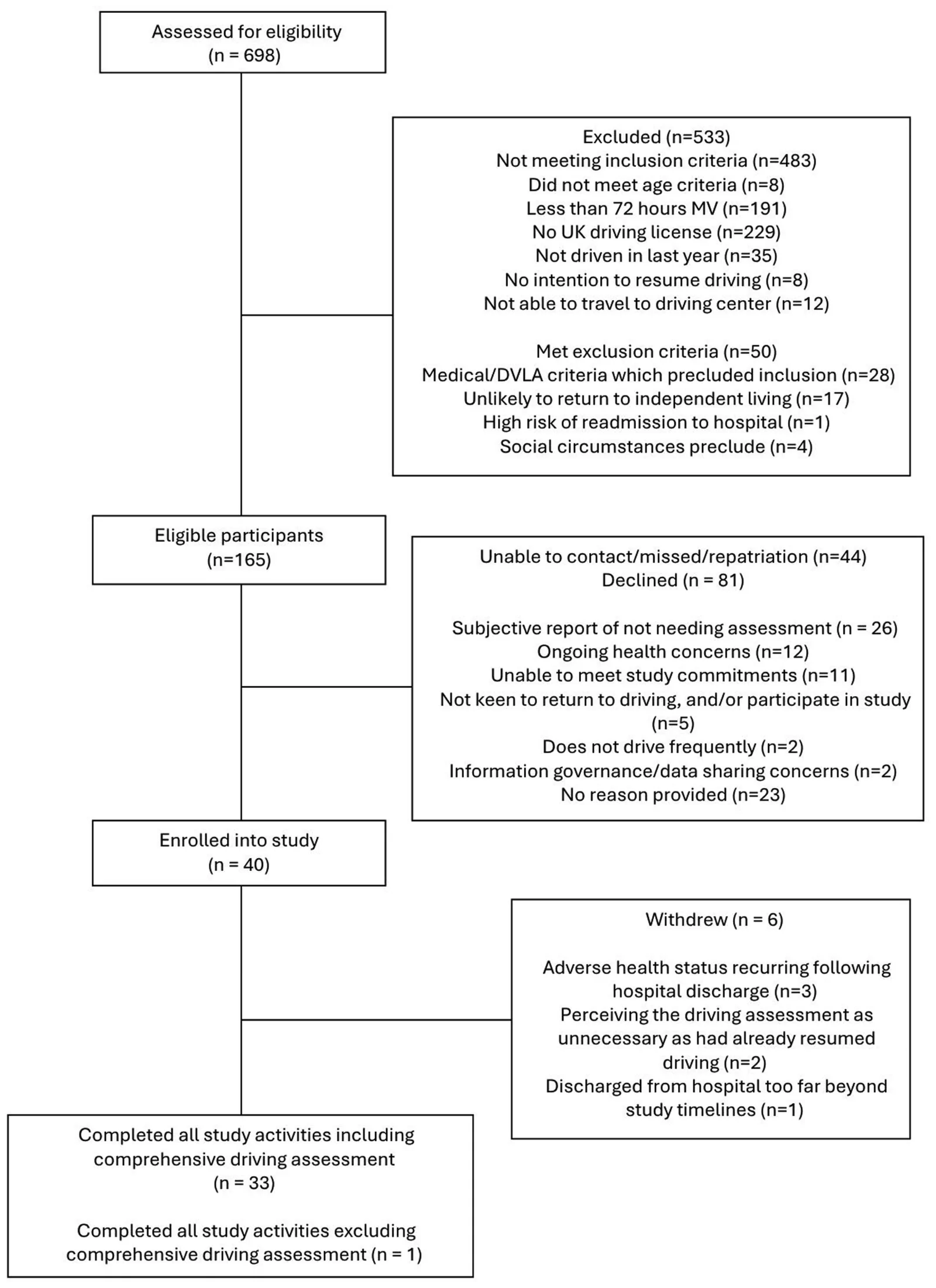
Consolidated Standards of Reporting Trials diagram. DVLA = Driver Vehicle Licensing Agency, MV = mechanical ventilation.

Participant characteristics are shown in **Table 1**. Median (IQR) time from hospital discharge to the comprehensive driving assessment was 12.5 weeks (10–18 wk). Nineteen participants (58%) had one or more positive finding detected on physical assessment; loss of range of movement (*n* = 8, 42%), reduced strength (*n* = 8, 42%), reduced coordination (*n* = 4, 21%), reduced proprioception (*n* = 5, 26%), and altered sensation (*n* = 11, 58%). Six participants (18%) had one or more positive finding from the cognitive battery; reduced speed of processing (*n* = 4, 12%), attention (*n* = 3, 9%), memory (*n* = 2, 6%), visuospatial perception (*n* = 2, 6%), and executive functioning (*n* = 1, 3%). Detail of the physical and cognitive assessments are shown in **Figure 2**.

**TABLE 1.** Participant Characteristics

*n* = 33	*n* (%)
Age, yr, median (IQR)	52 (46–57)
Female sex	10 (30)
ICU admission diagnosis	
Medical	21 (64)
Respiratory	13 (62)
Cardiovascular	5 (24)
Gastrointestinal	2 (10)
Neurological	1 (5)
Surgical	12 (36)
Gastrointestinal	4 (33)
Cardiothoracic	4 (33)
Vascular	4 (33)
Mechanical ventilation duration, d^a^, median (IQR)	10 (6–18)
Delirium^b^	14 (42)
Renal replacement therapy^c^	12 (36)
Acute Physiology and Chronic Health Evaluation II score^d,e^, median (IQR)	16 (13–22)
Sequential Organ Failure Assessment score^d,e^, median (IQR)	6 (4–8)
Extracorporeal membrane oxygenation	9 (27)
ICU LOS, d, median (IQR)	15 (11–33)
Hospital LOS, d, median (IQR)	47 (21–70)
Vehicle driven pre-admission	
Manual	18 (55)
Automatic	15 (45)
Employment status	
Full-time	22 (67)
Part-time	2 (6)
Retired	7 (21)
Not working	2 (6)
Living status	
Alone	5 (15)
With partner/spouse/family	28 (85)
Time to driving resumption, d, median (IQR)	72 (44–122)

IQR = interquartile range, LOS = length of stay.

a Missing data on three participants.

b Missing data on four participants.

c Missing data on three participants.

d Acute Physiology and Chronic Health Evaluation (APACHE) II and Sequential Organ Failure Assessment (SOFA) score for first 24 hr of ICU admission.

e Missing APACHE II and SOFA scores for five participants.

All data are *n* (%) unless otherwise specified.

**Figure 2.**
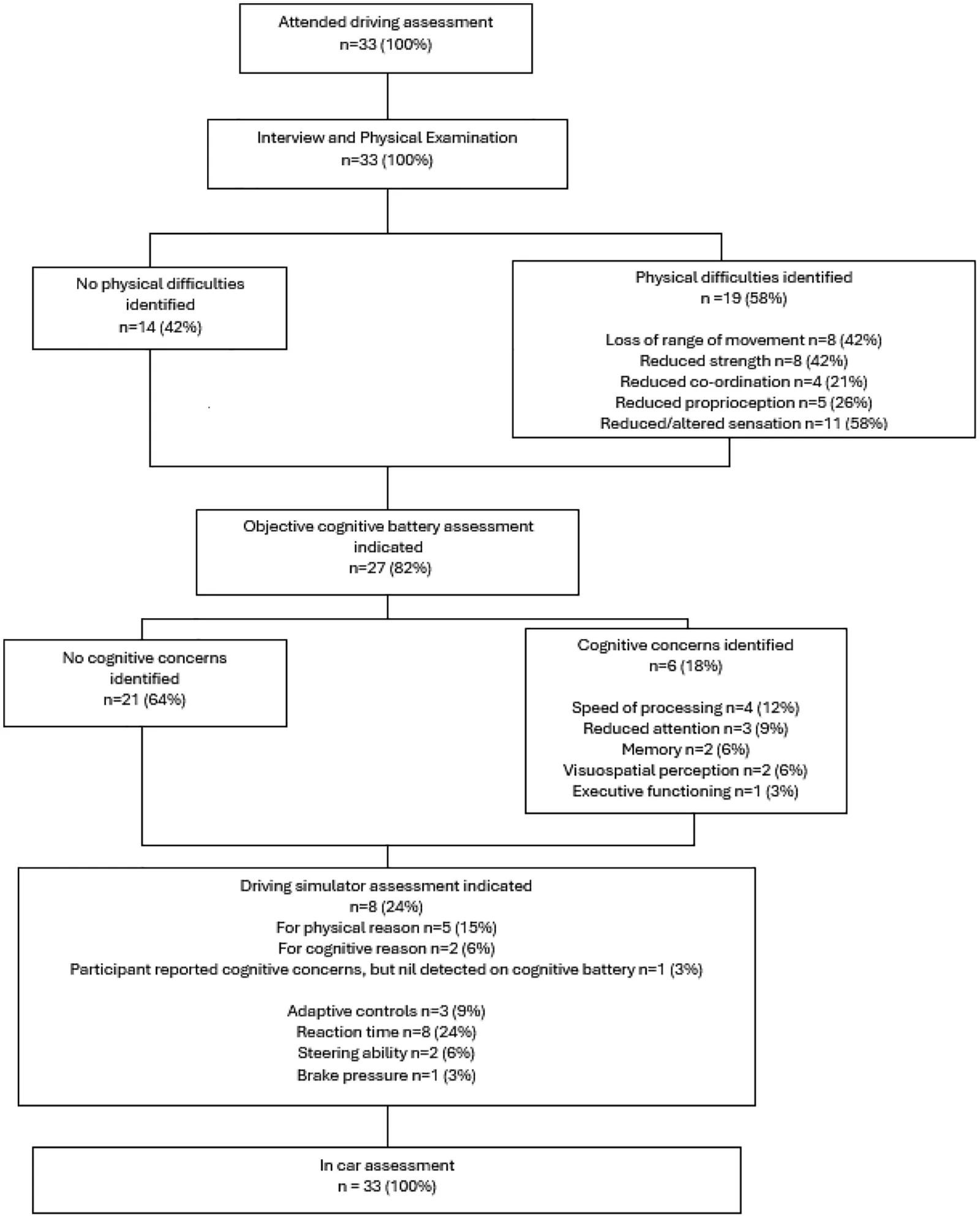
Participant completion of driving assessment components.

### Driving Ability

Twenty-six of the 33 participants (79%) who completed the comprehensive driving assessment were deemed able to drive a standard vehicle despite 21 (64%) having at least one positive finding on the physical or cognitive assessment. However, only 15 of 33 participants (45%) had resumed driving at the time of assessment. The remaining seven participants (21%) were deemed unable to drive due to a physical, psychological, or cognitive driving-related impairment. Physical driving-related impairments in four participants were loss of movement (*n* = 3, 9%) and loss of limb (*n* = 1, 3%), necessitating vehicle adaptations and further instruction on driving. Psychological impairments in the form of reduced confidence were identified in two participants (6%) necessitating refresher lessons in a standard vehicle. Cognitive impairment (reduced visuospatial awareness) was identified in one participant (3%) necessitating refresher lessons and a formal vision/hearing review. Four of the seven participants (57%) initially deemed unable to drive resumed driving successfully after the recommended specialist interventions during the 12-month study period.

Results from the cognitive assessment battery, driving simulator, and in-car assessment are included in **Supplementary Materials 3–5** (https://links.lww.com/CCM/I44). Driving-related impairments, recommended interventions, and subsequent driving resumption status are summarized in **Table 2**.

**TABLE 2.** Driving Ability and Driving-Related Impairments (*n* = 33)

Impairment Type	Diagnosis	*n* (%)	Method of Detection	Intervention	12-mo Outcome
None	—	26 (79)	—	—	Resumed driving (*n* = 25)
					Not resumed driving (*n* = 1)
Physical impairment	Spinal cord injury	1 (3)	Interview	Retrain with hand controls	Resumed driving
			Physical examination	Reassessment	
	Stroke	1 (3)	Interview	Steering wheel aid	Resumed driving
			Physical examination		
			Driving simulator		
	Lower limb amputation	1 (3)	Interview	Retrain with hand controls	Not resumed at 12 mo
			Physical examination		
	Distal lower limb dysfunction	1 (3)	Interview	Lower limb exercise program	Not resumed at 12 mo
			Physical examination	Reassess 12 mo	
				Retrain with left foot brake and accelerator	
Psychological impairment	Reduced confidence	2 (6)	Interview	Refresher lessons (*n* = 1)	Resumed driving (*n* = 1)
			In-car assessment	Reassessment (*n* = 1)	Unknown^a^ (*n* = 1)
Cognitive impairment	Visuospatial deficit	1 (3)	Cognitive battery	Refresher lessons	Resumed driving
			In-car assessment	Optician review	
				Hearing review	

a Patient did not complete 12-mo follow-up.

Interview comprised of subjective history including present condition, past medical history, drug history/current medications, driving history, and social situation. The dashes (—) indicate non applicable.

### Participant-Reported Driving Status

The proportion of participants reporting driving resumption at 3, 6, and 12 months after hospital discharge was 21 (62%), 28 (82%), and 30 (91%) participants, respectively. Median (IQR) time to driving resumption was 67 days (43–119 d; **Fig. 3**). Reasons for not resuming driving by 12 months (three participants) were: 1) ongoing lower limb impairment, 2) awaiting surgery for newly diagnosed ophthalmic issue, and 3) social factors and expense.

**Figure 3.**
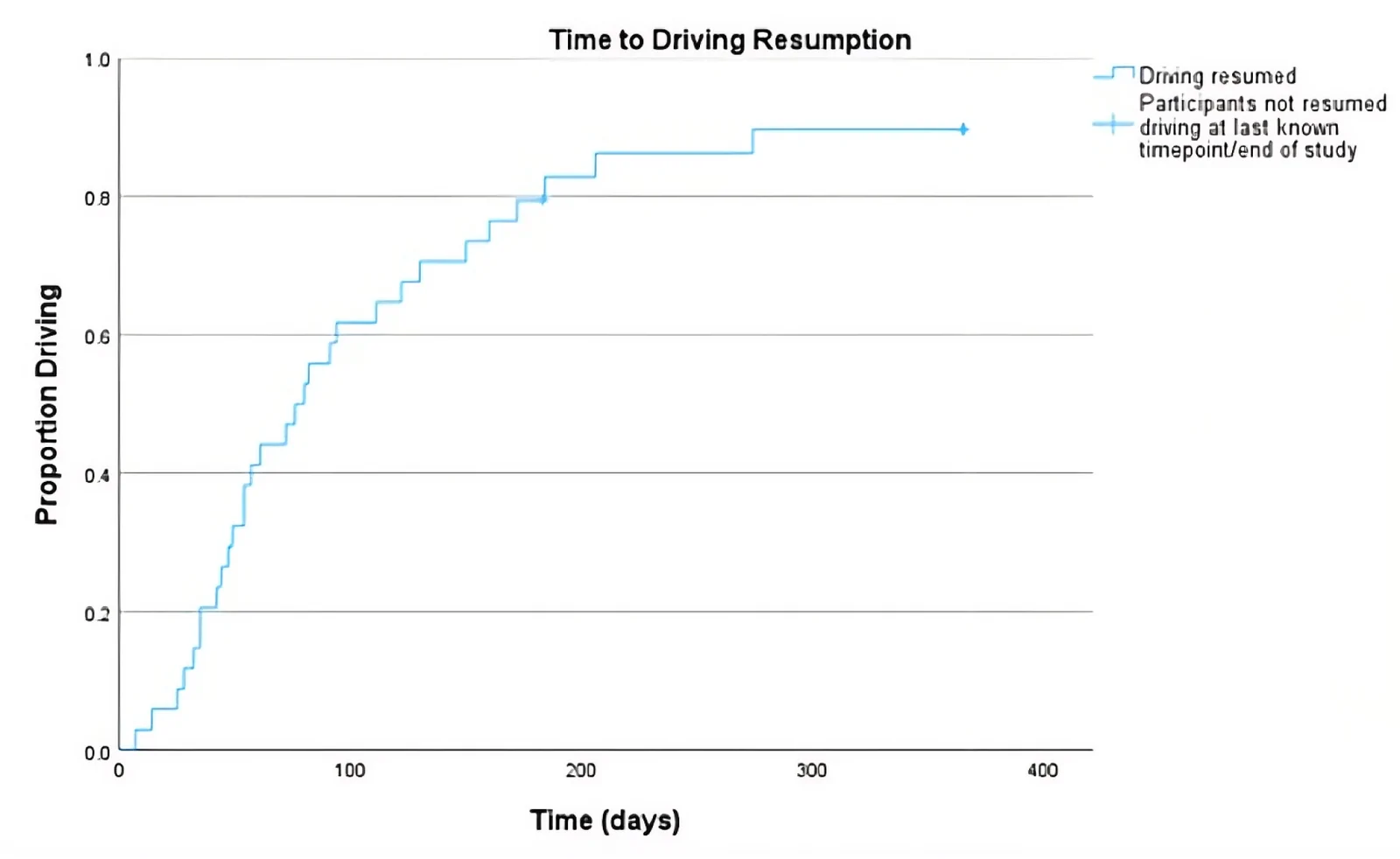
Time to driving resumption.

Self-reported reasons for not resuming driving in the period before the driving assessment (18 participants) were: physical/mobility concerns (*n* = 12, 67%), no need to drive (*n* = 8, 44%), reduced confidence (*n* = 6, 33%), anxiety (*n* = 5, 28%), family member concerns (*n* = 3, 17%), unaware driving resumption was an option (*n* = 3, 17%), tiredness/fatigue (*n* = 2, 11%), thinking/memory concerns (*n* = 1, 6%), concentration concerns (*n* = 1, 6%), and eyesight concerns (*n* = 1, 6%). Median (IQR) driving confidence score (self-report 0–10 scale) after the driving assessment was 9 (7–10) compared with 7 (5–8) preceding the assessment (*p* < 0.05).

Of the 15 participants who had already resumed driving before the assessment, 13 (87%) had driven four or more times. Despite driving resumption, participants reported physical differences (*n* = 5) including dizziness, visual changes, weakness, pain, and fatigue, reduced confidence (*n* = 2), reduced reaction times (*n* = 1), mood changes (*n* = 1), and concerns about driving capability (*n* = 1).

No prior driving guidance or advice was received by 24 participants (73%), whereas nine participants (27%) recalled receiving information on driving either from their general practitioner (GP), critical care recovery services, hospital team, the DVLA, and/or internet sources. Two participants (6%) reported self-assessing readiness to resume driving, referencing confidence and energy levels.

### Acceptability and Perception of the Driving Assessment

Thirty-two participants (97%) rated the acceptability of the formal driving assessment as very good or excellent. Reported benefits of driving assessment completion were: reassurance of skills/driving capability (*n* = 16, 48%), improvement in confidence (*n* = 13, 39%), ensuring driving safety (*n* = 5, 15%), reducing anxiety (*n* = 2, 6%), promoting independence (*n* = 1, 3%), and empowerment (*n* = 1, 3%).

## DISCUSSION

Using a comprehensive formal driving assessment approximately 3 months following hospital discharge, we found that, despite a high proportion of participants having positive findings on the physical and cognitive assessments, around three-quarters were deemed able to drive a standard vehicle without requiring any further driving support. Of those unable to drive due to a physical, psychological, or cognitive impairment, over half were able to resume driving after completing driving enablement interventions (such as vehicle adaptations, retraining, or refresher lessons) identified by the formal driving assessment. While only two thirds had resumed driving at 3 months, nearly all participants had resumed driving at 12 months. Few participants received guidance from any source (hospital, primary care team/GP) regarding driving resumption. Completing the driving assessment improved participant competence in driving ability and safety.

Approximately half of the participants deemed able to drive had not resumed driving before the driving assessment primarily due to physical/mobility concerns and lack of confidence. Participants reported increased confidence, reduced anxiety, and reassured family members after the driving assessment. Furthermore, participants rated the acceptability of the comprehensive driving assessment as very good to excellent and valued the opportunity to be assessed by driving experts. Therefore, a formal driving assessment can be both a “diagnostic” and “therapeutic” intervention, enhancing safety and reducing unwarranted driving delay by addressing common subjective driving barriers.

In the United Kingdom, comprehensive driving assessments are considered the gold standard in assessing fitness to drive (23–25) with studies completed in dementia, cerebrovascular accident, and older age populations but never previously studied in critically ill adults (26, 27). This is one of the first studies to prospectively evaluate driving ability using a comprehensive driving assessment in a cohort of individuals recovering from critical illness. Findings from a study investigating driving resumption in critical illness survivors using in-vehicle sensors are awaited (ClinicalTrials.gov ID: NCT05999903).

In this cohort, the cognitive assessment battery did not forecast on-road driving performance. The cognitive assessment battery used in this study was based on the Rookwood Driving Battery, which is designed to assess cognitive fitness to drive in neurological populations (28). One purpose of the battery is to screen for individuals who would fail an on-road test because of a cognitive deficit; however, results should be considered in combination with other evidence (17). The validity of the measure in predicting the outcome of on-road tests in certain health conditions, including dementia and multiple sclerosis, is debated among the literature (18, 19, 28, 29). The validity of the measure in older adults has also been raised (30). The battery has yet to be validated in the critical care population. Not all tests in the Rookwood Driving Battery were included in the cognitive assessment battery utilized with our participants; however, in our cohort of critical illness survivors, cognitive testing was not a valid surrogate for an on-road assessment.

Worldwide, an estimated 51% of journeys are completed by car, with variations noted across different continents, regions, and cities (31). In North America and Australasia, car use for commuting is 92% and 76%, respectively, compared with 45% in Europe (31). Driving is deemed essential for work, education, healthcare (32–34), the resumption of social activities and family roles (5, 6), especially in rural communities or where public transportation is deficient (6). In our study, around three quarters of participants could have resumed driving earlier than they did suggesting a role for driving enablement interventions not just determination of eligibility. The lack of guidance on driving resumption both nationally and internationally may be due to a paucity of evidence to base recommendations. Currently, there is no comprehensive review identifying international differences to driving after critical illness. We intend for the findings of this study to inform future work and evidence based guidelines, which could be disseminated via medical professional bodies and country-specific road safety authorities.

The prospective design, collaboration with a specialist DVLA-approved driving center, and a comprehensive formal driving assessment delivered by specialist approved driving instructors and driving-specialized OTs, including in-car and on-road assessments, are strengths of this study.

The limitations of our study include a relatively small sample size meaning our results may not be generalizable to other critical care populations. The voluntary nature of participation likely introduced selection bias from people particularly interested in resuming driving opting to participate. Not every participant completed the comprehensive driving assessment at the planned 2-month time point owing to participant and appointment availability. Rating of driving assessment acceptability and driving confidence may have been influenced by circumstantial factors, that is, data collection in a location with proximity to the driving assessment center by a member of the research team. Cost of the driving assessment and associated travel to the driving assessment center were covered by the study; however, we recognize this may be a barrier for self-paying individuals or those with lower economic resource. Our study did not incorporate patient-reported outcome measures recommended in recent guidance, nor road traffic collision data, which could have provided valuable insights (35, 36). However, it remains unclear whether impairments identified through the recommended measures identify patients not deemed safe for driving.

## CONCLUSIONS

In adults recovering from critical illness, functional or cognitive impairments prohibiting driving, as identified by a comprehensive formal driving assessment, were low despite most of the cohort having at least one positive finding on physical or cognitive assessment. Most participants assessed as unable to drive were able to resume driving with vehicle adaptations, retraining, and/or refresher lessons. Despite under half of the participants having resumed driving by the driving assessment time point, the proportion deemed safe to drive far exceeded this. Further research is warranted on the role of early post-ICU driving assessment and to establish evidence based guidance to support safe and timely return to driving in individuals recovering from critical illness.

## ACKNOWLEDGMENTS

We thank all the patients who participated, the staff at QEF Mobility Services, research staff at the participant identification centers, and the post-ICU recovery service team at Guy’s and St Thomas’ National Health Service Foundation Trust.

## Supplementary Material
