# Supplementary material for: Comprehensive Driving Assessment to Enable Driving Resumption in Adults Recovering From Critical Illness: A Prospective Cohort Study
Source: Crit Care Med. 2026 Sep 11;54(11):2856–66. doi: 10.1097/CCM.0000000000007316 (PMC13722437; doi:10.1097/CCM.0000000000007316)
Supplement: Supplementary file 1 [file ccm-54-2856-s001.pdf]

Supplementary Material 1: Cognitive Assessment Battery

Executive Functioning

Weigl Sorting Test:

Assesses problem solving ability. Requires the recognition of colour and shape as dimensions for grouping a set of 12 shapes

Maximal score is 4. Good = 4, Borderline = 3, Poor = 0-2.

Comprehension Token Test:

Assesses receptive language ability. Makes use of the shapes of the Weigl Sorting Test and the individual is asked to move the shapes according to instructions.

Maximal score is 8, Good = 7-8, Borderline = 6, Poor = 0-5.

Card Rule Shift:

Assesses change of concept ability

Maximal score is 20, Good = 20, Borderline = 18-19, Poor = 0-17.

Tapping and sequence of hand movements:

Assesses self-monitoring of an intended movement and orchestrating a new movement.

Tapping requires the individual to produce a simple movement (one or two taps) in response to the tapping produced by the assessor. Sequencing requires the individual has to learn a simple sequence of three hand movements.

Maximal score is 15, Good = 14-15, Borderline =13, Poor = 0-12.

Perceptual Analysis

Letter Cancellation

Assesses visual scanning ability. Participants are given 100 seconds to find and mark target letters within a larger array of letters.

Maximal score is 86, 42 or less = Poor, 5 or more errors = Fail.

Cube Analysis:

Assesses visual/spatial appreciation. Requires participants to determine how many bricks have been used to create a 3D arrangement, represented by a two-dimensional drawing.

Maximal score is 10, Good = 10, Borderline = 8-9, Poor = 0-7.

Position Discrimination:

Assesses visual awareness/spatial appreciation. Participants are shown two squares with a dot inside each and asked to determine which dot is placed in the exact centre of the square.

Maximal score is 20, Good = 19-20, Borderline = 17-18, Poor = 0-16.

Shape Perception:

Assesses visual memory ability

Maximal score is 20, Good = 19-20, Borderline = 18, Poor = 0-17.

Divided Attention:

Assesses multitasking/attention. This combines a retest of the letter cancellation test with an audio presentation of a pre-recorded story. The participant must cancel the letters while also marking on the sheet every time the audio says the word "three".

Maximal score is 86, 36 or less = Poor, 5 or more errors = Fail.

Supplementary Material 2: Driving Simulator Assessment

Traffic light system used for assessment. Green = Good/ within normal range, Amber = Outside of normal range, participant may need adaptations, Red = Assistance/adaptations required.

Static steering strength (seconds)

Criteria: Less than 4 seconds = Green, 4-6 seconds = Amber, Over 6 seconds = Red

Brake Pressure (Newtons) – Pedal Controls

Criteria: Average force over 340N = Green, Average force of 140-340N = Amber, Average force of less than 140N = Red

Reaction times (seconds)

Criteria: Average reaction time of less than 1 second = Green, Average reaction time of 1.0-1.25 seconds = Amber, Reaction time of over 1.25 seconds = Red

Decision making times (seconds)

Criteria: Average decision time of less than 1 second = Green, Average reaction time of 1.0-1.25 seconds = Amber, Decision time of over 1.25 seconds = Red

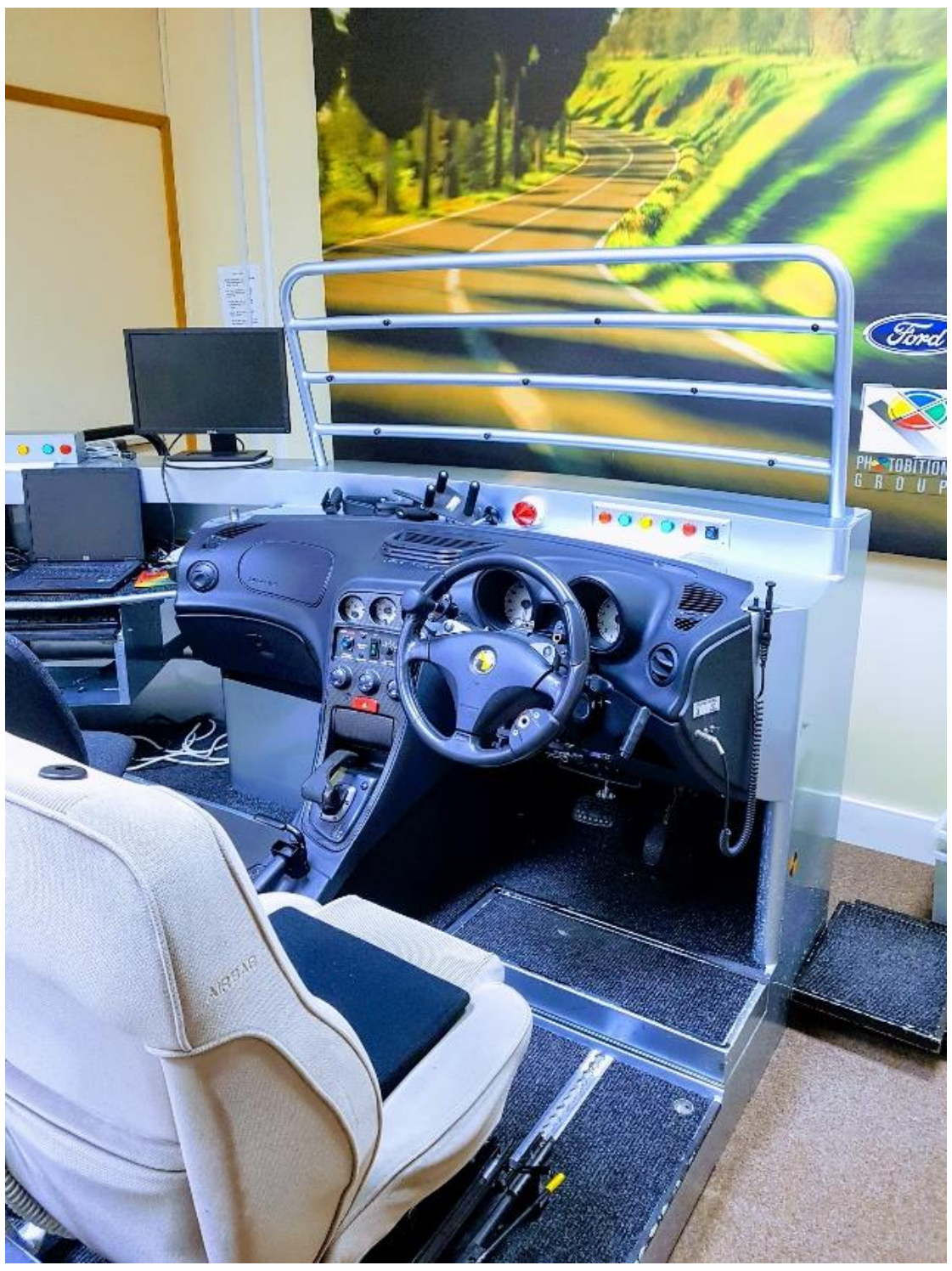

Supplementary Material Table 3: Cognitive Assessment Battery

Cognitive Assessment	Outcome from Cognitive Assessment N (%)		
	Good	Borderline	Poor
Executive Functioning			
Weigl Sorting Test (n=10)	10 (100)	-	-
Comprehension Token Test (n=10)	9 (90)	-	1 (10)
Card Rule Shift (n=24)	20 (83)	4 (17)	-
Tapping and sequence of hand movement (n=7)	6 (86)	1 (14)	-
Perceptual Analysis			
Letter Cancellation (n=18)	18 (100)	-	-
Cube Analysis (n=22)	19 (86)	2 (9)	1 (5)
Position Discrimination (n=22)	20 (90)	1 (5)	1 (5)
Shape Perception (n=21)	20 (95)	1 (5)	-
Divided Attention (n=16)	16 (100)	-	-

Note: Weigl Sorting Test, maximal score is 4. Good = 4, Borderline = 3, Poor = 0-2. Comprehension Token Test, maximal score is 8, Good = 7-8, Borderline = 6, Poor = 0-5. Card Rule Shift, maximal score is 20, Good = 20, Borderline = 18-19, Poor = 0-17. Tapping and sequence of hand movements, maximal score is 15, Good = 14-15, Borderline =13, Poor = 0-12. Letter Cancellation, maximal score is 86, 42 or less = Poor, 5 or more errors = Fail. Cube Analysis maximal score is 10, Good = 10, Borderline – 8-9, Poor = 0-7. Position Discrimination, maximal score is 20, Good = 19-20, Borderline = 17-18, Poor = 0-16. Shape Perception, maximal score is 20, Good = 19-20, Borderline = 18, Poor = 0-17. Divided Attention, maximal score is 86, 36 or less = Poor, 5 or more errors = Fail.

Supplementary Material Table 4: Driving simulator assessment

N=8	Driving simulator						
	Controls	Static steering strength (seconds)	Brake Pressure (Newtons)	Reaction times (seconds)	Reaction times safety criteria met	Decision making times (seconds)	Decision making times safety criteria met
Participant 1	Standard	N/A	N/A	0.52	Yes	0.99	Yes
Participant 2	Standard	Right turn with both hands - Knob position 2 = 3.80<br>Right turn with both hands – Knob position 3 = 4.45<br>Right turn with steering aid – Knob position 2 = 1.95<br>Left turn with steering aid – Knob position 2 = 1.75<br>Left turn with both hands – Knob position 2 = 3.04<br>Left turn with both hands – Knob position 3 = 3.43	N/A	0.6	Yes	N/A	N/A
Participant 3	Accelerator - right hand push pull leaver<br>Brake – right hand	N/A	N/A	0.56	Yes	N/A	N/A
Participant 4	Accelerator – right hand push-pull level.<br>Brake – right hand – push pull leaver	N/A	N/A	0.52	Yes	N/A	N/A
Participant 5	Standard	N/A	N/A	0.58	Yes	N/A	N/A
Participant 6	Right foot brake and accelerator.<br>Left foot brake and accelerator<br>Hand controls	N/A	N/A	Right foot brake and accelerator = 0.72<br>Left foot brake and accelerator = 0.79<br>Hand controls = 0.46	Yes	N/A	N/A
Participant 7	Standard	Left turn with both hands - Knob position 0 - total time= 2.48<br><br>Right turn with both hands – Knob position 0 – total time = 2.68	Average = 339.82	0.54	Yes	N/A	N/A
Participant 8	Standard	N/A	N/A	0.48	Yes	N/A	N/A

Supplementary Material Table 5: In car driving assessment metrics

N=31*	Driving evaluation score n (%)				
Driving component	1	2	3	4	Not applicable/ not assessed
Practical skills (n=31)*					
Steering	28 (90)	3 (10)	0	0	0
Accelerator	25 (81)	5 (16)	1 (3)	0	0
Brake	26 (74)	5 (16)	0	0	0
Clutch	17 (55)	2 (6)	0	0	12 (39)
Gears	29 (94)	2 (6)	0	0	0
Parking Brake	29 (94)	2 (6)	0	0	0
Secondary controls	28 (90)	2 (6)	1 (3)	0	0
Observation, Mirrors, Blind spot	25 (81)	5 (16)	1 (3)	0	0
Higher driving skills					
Sequencing and co-ordination	27 (87)	2 (6)	1 (3)	0	1 (3)
Visuospatial perception	25 (81)	4 (13)	1 (3)	0	1 (3)
Attention	24 (77)	6 (19)	0	0	1 (3)
Memory	29 (94)	1 (3)	0	0	1 (3)
Planning	23 (74)	7 (23)	0	0	1 (3)
Speed of Processing	25 (81)	5 (16)	0	0	1 (3)
Executive Function	24 (77)	6 (19)	0	0	1 (3)

Note: *Two participants who trialled new vehicle controls were not evaluated using this metric. Scoring system: 1 = Good, 2 = Acceptable, 3 = Poor, 4 = High Risk. Scores of 1 and 2 were considered to represent safe driving. One participant only completed a short drive due to reduced confidence so was not assessed/scored on higher driving skills.

Supplementary Material Table 6: Characteristics of enrolled participants

N = 40	n (%)
Age, years, median IQR	53 (46, 64)
Female sex	13 (32)
ICU admission diagnosis	
Medical	24 (60)
Respiratory	15 (63)
Cardiovascular	5 (21)
Gastrointestinal	2 (8)
Neurological	2 (8)
Surgical	16 (40)
Gastrointestinal	6 (38)
Cardiothoracic	5 (31)
Vascular	5 (31)
Invasive ventilation duration, days[a], median IQR	9 (6, 17)
Delirium[b]	16 (40)
Renal replacement therapy[c]	16 (40)
APACHE II score,[d,e] median IQR	18 (13, 22)
SOFA score[d,e] median IQR	6 (4, 8)
ECMO	9 (23)
ICU LOS, days, median IQR	15 (11, 32)
Hospital LOS, days, median IQR	41.5 (21, 68)
Vehicle driven pre-admission	
Manual	18 (45)
Automatic	15 (38)
Unknown[f]	7 (17)
Employment status	
Full-time	24 (60)
Part-time	3 (8)
Retired	10 (25)
Not working	2 (5)
Unknown	1 (3)
Living status	
Alone	6 (15)
With partner/spouse/family	33 (83)
Unknown	1 (3)
Time to driving resumption, days, median IQR	67 (43, 119)

a Missing data on four participants
b Missing data on five participants
c Missing data on four participants
d APACHE II and SOFA score for 1st 24 hours of ICU admission
e Missing APACHE II and SOFA scores for six participants
f Participants did not complete driving assessment thus vehicle type unknown
All data are n (%) unless otherwise specified.
APACHE: Acute Physiology and Chronic Health Evaluation; ECMO: Extracorporeal Membrane Oxygenation; ICU: Intensive Care Unit; LOS: Length of stay; SOFA: Sequential Organ Failure Assessment

Supplementary Material 7: STROBE Statement—Checklist of items that should be included in reports of cohort studies

	Item No	Recommendation	Page No
**Title and abstract**	1	(*a*) Indicate the study's design with a commonly used term in the title or the abstract	1
		(*b*) Provide in the abstract an informative and balanced summary of what was done and what was found	5, 6
**Introduction**			
Background/rationale	2	Explain the scientific background and rationale for the investigation being reported	8
Objectives	3	State specific objectives, including any prespecified hypotheses	9
**Methods**			
Study design	4	Present key elements of study design early in the paper	9
Setting	5	Describe the setting, locations, and relevant dates, including periods of recruitment, exposure, follow-up, and data collection	9
Participants	6	(*a*) Give the eligibility criteria, and the sources and methods of selection of participants. Describe methods of follow-up	9
		(*b*) For matched studies, give matching criteria and number of exposed and unexposed	N/A
Variables	7	Clearly define all outcomes, exposures, predictors, potential confounders, and effect modifiers. Give diagnostic criteria, if applicable	10
Data sources/ measurement	8*	For each variable of interest, give sources of data and details of methods of assessment (measurement). Describe comparability of assessment methods if there is more than one group	10, 11
Bias	9	Describe any efforts to address potential sources of bias	10
Study size	10	Explain how the study size was arrived at	–
Quantitative variables	11	Explain how quantitative variables were handled in the analyses. If applicable, describe which groupings were chosen and why	11, 12
Statistical methods	12	(*a*) Describe all statistical methods, including those used to control for confounding	12
		(*b*) Describe any methods used to examine subgroups and interactions	N/A
		(*c*) Explain how missing data were addressed	12
		(*d*) If applicable, explain how loss to follow-up was addressed	12
		(*e*) Describe any sensitivity analyses	N/A
**Results**			
Participants	13*	(a) Report numbers of individuals at each stage of study—eg numbers potentially eligible, examined for eligibility, confirmed eligible, included in the study, completing follow-up, and analysed	12, Fig 1
		(b) Give reasons for non-participation at each stage	Fig 1
		(c) Consider use of a flow diagram	Fig 1
Descriptive data	14*	(a) Give characteristics of study participants (eg demographic, clinical, social) and information on exposures and potential confounders	Table 1
		(b) Indicate number of participants with missing data for each variable of interest	Table 1
		(c) Summarise follow-up time (eg, average and total amount)	12, 13,
Outcome data	15*	Report numbers of outcome events or summary measures over time	13, Tables 2, 3,4, 5

Supplementary Material 8: The TIDieR (Template for Intervention Description and Replication) Checklist

Item number	Item	Where located **	
		Primary paper (page or ap number)	Other † (details)
	**BRIEF NAME**		
1.	Provide the name or a phrase that describes the intervention.	1	______________
	**WHY**		
2.	Describe any rationale, theory, or goal of the elements essential to the intervention.	9, 10	_____________
	**WHAT**		
3.	Materials: Describe any physical or informational materials used in the intervention, including those provided to participants or used in intervention delivery or in training of intervention providers. Provide information on where the materials can be accessed (e.g. online appendix, URL).	11	_____________
4.	Procedures: Describe each of the procedures, activities, and/or processes used in the intervention, including any enabling or support activities.	9, 10, 11	_____________
	**WHO PROVIDED**		
5.	For each category of intervention provider (e.g. psychologist, nursing assistant), describe their expertise, background and any specific training given.	10, 11	_____________
	**HOW**		
6.	Describe the modes of delivery (e.g. face-to-face or by some other mechanism, such as internet or telephone) of the intervention and whether it was provided individually or in a group.	10	_____________
	**WHERE**		
7.	Describe the type(s) of location(s) where the intervention occurred, including any necessary infrastructure or relevant features.	10	_____________
	**WHEN and HOW MUCH**		
8.	Describe the number of times the intervention was delivered and over what period of time including the number of sessions, their schedule, and their duration, intensity or dose.	10, 11	_____________
	**TAILORING**		
9.	If the intervention was planned to be personalised, titrated or adapted, then describe what, why, when, and how.	10, 11	_____________
	**MODIFICATIONS**		

**10.**‡	If the intervention was modified during the course of the study, describe the changes (what, why, when, and how).	N/A	____________
	**HOW WELL**		
**11.**	Planned: If intervention adherence or fidelity was assessed, describe how and by whom, and if any strategies were used to maintain or improve fidelity, describe them.	N/A	____________
**12.**‡	Actual: If intervention adherence or fidelity was assessed, describe the extent to which the intervention was delivered as planned.	12	Figure 1
